# SEOM Clinical Guideline update for the prevention of chemotherapy-induced nausea and vomiting (2021)

**DOI:** 10.1007/s12094-022-02802-1

**Published:** 2022-03-26

**Authors:** Margarita Majem, Ramon de las Peñas, Juan Antonio Virizuela, Luís Cabezón-Gutiérrez, Patricia Cruz, Rafael Lopez-Castro, Miriam Méndez, Rebeca Mondéjar, María del Mar Muñoz, Yolanda Escobar

**Affiliations:** 1 Department of Medical Oncology, Hospital de la Santa Creu i Sant Pau, c/Sant Antoni Maria Claret 167, 08025 Barcelona, Spain; 2 Department of Medical Oncology, Hospital Provincial Castelló, Valencia, Spain; 3 Department of Medical Oncology, Hospital Universitario Virgen Macarena, Seville, Spain; 4 Department of Medical Oncology, Hospital Universitario de Torrejón, Madrid, Spain; 5 Department of Medical Oncology, Hospital Universitario La Paz, Madrid, Spain; 6 Department of Medical Oncology, Hospital Clínico Universitario de Valladolid, Valladolid, Spain; 7 Department of Medical Oncology, Hospital Universitario Puerta de Hierro-Majadahonda, Madrid, Spain; 8 Department of Medical Oncology, Hospital Universitario de La Princesa, Madrid, Spain; 9 Department of Medical Oncology, Hospital Virgen de la Luz de Cuenca, Cuenca, Spain; 10 Department of Medical Oncology, Hospital General Universitario Gregorio Marañón, Madrid, Spain

**Keywords:** Emesis, Nausea, Vomiting, Chemotherapy

## Abstract

Among the side effects of anticancer treatment, chemotherapy-induced nausea and vomiting (CINV) is one of the most feared given its high prevalence, affecting up to 40% of patients. It can impair patient’s quality of life and provoke low adherence to cancer treatment or chemotherapy dose reductions that can comprise treatment efficacy. Suffering CINV depends on factors related to the intrinsic emetogenicity of antineoplastic drugs and on patient characteristics. CINV can appear at different times regarding the administration of antitumor treatment and the variability of risk according to the different antitumor regimens has, as a consequence, the need for a different and adapted antiemetic treatment prophylaxis to achieve the desired objective of complete protection of the patient in the acute phase, in the late phase and in the global phase of emesis. As a basis for the recommendations, the level of emetogenicity of anticancer treatment is considered and they are classified as high, moderate, low and minimal emetogenicity and these recommendations are based on the use of antiemetic drugs with a high therapeutic index: anti 5-HT, anti-NK and steroids. Despite having highly effective treatments, clinical reality shows that they are not applied enough, so evidence-based recommendations are needed to show the best options and help in decision-making. To cover all the antiemetic prophylaxis options, we have also included recommendations for oral treatments, multiday regimens and radiation-induced emesis prevention.

## Introduction

Among the side effects of anticancer treatment, chemotherapy-induced nausea and vomiting (CINV) is one of the most feared by patients given its high prevalence, affecting up to 40% of patients [1]. It can impair patient’s quality of life [2], and also provoke low adherence to cancer treatment [3] or chemotherapy dose reductions that can compromise treatment efficacy [4].

The likelihood of suffering from CINV weighs down, on one hand, on patient conditioning factors and, on the other hand, on intrinsic cancer drug properties.

Some patient characteristics are linked with higher emesis related with cancer treatment: low performance status, younger age, female gender, unusual alcohol intake, hyperemesis gravidarum and motion sickness; also, medical conditions such as previous CINV, anxiety, metabolic disorders (dehydration, hyperkalemia, hypocalcemia, hyponatremia), ascites, bowel obstruction, and use of concurrent drugs (opioids, antibiotics…) can increase this risk [5–7]. There can also be a genetic predisposition through polymorphisms of the enzymes that metabolize 5-HT3 receptor antagonists and of the receptor itself that increases emesis probabilities [8].

Regarding cancer drugs, their own characteristics and risk of CINV have led to classify them into four groups regarding their probability of emesis: highly, moderately, low- and minimallyemetogenic drugs; their combination in polychemotherapy regimens also increases the risk (Tables 1 and 2) [9].

**Table 1:** Emetogenic potential of parenteral anticancer agents

Level	Agent			
High emetic risk (> 90% frecuency of emesis)	AC combination defiend as any chemotherapy regimen that contains an antracyclinne and cyclophosphamide Carboplatin AUC ≥4 Carmustine > 250mg/m²	Cisplatin Cyclophosphamide> 1500mg/m² Dacarbazine Doxorubicine ≥60mg/m² Epirubicin> 90mg/m² Ifosfamide ≥2g/m² per dose	Meclorethamine Melphalan ≥ 140mg/m² Sacituzumab govitecan-hziy Streptozocin	
Moderate emetic risk (> (30–90% frecuency of emesis)	Aldesleukin > 12–15million IU/m² Amifostine > 300mg/m² Amivantamab Azacitidine Bendamustine Busulfán Carboplatino AUC <4 Carmustine ≤250mg/m² Clofarabine Cyclophosfamide ≤ 1500mg/m² Cytarabine> 200mg m² Dactinomicine	Daunorubicin Dual-drug liposomal encapsulation of cytarabine and daunorubicin Dinutuximab Doxorubicin <60mg/m² Epirubicin ≤90mg m² Fam-trastuzumab deruxtecan-nxki Idarubicin Ifosfamide <2g(m² per dose Irinotecan Irinotecan (liposomal)	Lurbinectedin Melphalan <140mg/m² Methotrexate ≥ 250mg/m² Naxitamab-gqgk Oxaliplatin Temozolomide Trabectedin	
Low emetic risk (10–30% frecuency of emesis)	Ado-trastuzumab emtansine Aldesleukin ≤12million IU/m² Amifostine < 300mg/m² Arsenic trioxide Axicabtagene ciloleucel Belinostat Brexucabtagene autoleucel Brentuximab vedotin Cabazitaxel Carfilzomib Cytarabine (low dose)100–200mg/m²	Docetaxel Doxorubicin (liposomal) Enfortumab vedotin-ejv Eribulin Etopóside 5-fluorouracil (5-FU) Floxuridine Gemcitabine Gemtuzumab ozogamicin Idecabtagene vicleucel Inotuzumab ozogamicina Isatuximab-irfc Ixabepilone lisocabtagene maraleucel Loncastuximab terisine-lpyl	Methotrexate > 50mg/m² < 250mg/m² Mitomycin Mitomycine pyelocaelyceal solution Mitoxantrone Mogamulizumab-kpkc Moxetumomab pasudotox-tdfk Necitumumab Omacetaxine Paclitaxel Paclitaxel-albumin Pemetrexed Pentostatina Polatuzumab vedotin	Pralatrexatee Tafasitamab-cxix Tagraxofusp-erzs Talimogene laherparepvec Thiotepa Tisagenlecleucel Tisotumab vendotin- Topotecán Ziv-aflibercept
Minimal emetic risk (<10% frecuency of emesis)	Alemtuzumab Asparaginase Atezolizumab Avelumab belantamab mafodotin-blmf Bevacizumab Bleomycin Blinatumomab Bortezomib Cetuximab Cemiplimab-rwlc Cladribine Cytarabine <100mg/m² Daratumumab	Daratumumab and hyaluronidase-fihj Decitabine Denileukin diftitox Dostarlimab-gxly Dexrazoxane Durvalumab Elotuzumab Fludarabine Ipilimumab Luspatercept-aamt Margetuximab-cmkb Methotrexate ≤50mg/m² Nelarabine Nivolumab	Obinutuzumab Ofatumumab Panitumumab Pembrolizumab Pertuzumab Pertuzumab / trastuzumab and hyaluronidase-zzxf Ramucirumab Rituximab Rituximab and hyaluronidase	Siltuximab Temsirolimus Trastuzumab Trastuzumab and hyaluronidase-oysk Valrubicin Vinblastine Vincristine Vincristine (liposomal) Vinorelbine

Based in NCCN clinical practice guidelines in oncology (NCCN Guidelines^®^) Antiemesis Version 1.2022

**Table 2:** Emetogenic potential of oral anticancer agents

Level	Agent			
Moderate to high emetic risk (≥30% frecuency of emesis)	Altretamine Avapritinib Azacytidine Binimetinib Bosutinib > 400mg/day Busulfan ≥ 4mg/day Cabozantinib Ceritinib	Crizotinib Cyclophosfamide ≥100 mg/m²/day Dabrafenib Enasidenib Encorafenib Estramustine	Etoposide Fedratinib Imatinib> 400mg/day Lenvatinib> 12mg/day Lomustine (single day) Midostaurin Mitotane Mobocertinib	Niraparib Olaparib Procarbazine Rucaparib Selinexor Temozolomide > 75mg/m²/day
Minimal to low emetic risk (<30% frecuency of emesis)	Abemaciclib Acalabrutinib Afatinib Alectinib Alpelisib Asciminib Axitinib Belzutifan Bexarotene Brigatinib Bosutinib ≤ 400mg/day Busulfan <4mg/day Capecitabine Capmatinib Chlorambucil Cobimetinib Cyclophosfamide <100mg/m²/day Dacomitinib Dasatinib Decitabine and cedazuridine	Duvelisib Entrectinib Erdafitinib Erlotinib Everolimus Fludarabine Gefitinib Gilteritinib Glasdegib Hydroxyurea Ibrutinib Idelalisib Imatinib ≤ 400mg/day Ixazomib Ivosidenib Lapatinib Larotrectinib Lenalidomide Lenvatinib ≤12mg/day	Lorlatinib Melphalan Mercaptopurine Methotrexate Nilotinib Neratinib Osimertinib Palbociclib Pazopanib Pemigatinib Pexidartinib Pomalidomide Ponatinib Pralsetinib Regorafenib Ribociclib Ripretinib Ruxolitinib Selpercatinib	Sonidegib Sorafenib Sotorasib Sunitinib Talazoparib tosylate Tazemetostat Temozolomide ≤75mg/m²/day Tepotinib Thalidomide Thioguanine Tivozanib Topotecán Trametinib Tretinoin Trifluridine/tipiracil Tucatinib Umbralisib Vandetanib Vemurafenib Venetoclax Vismodegib Vorinostat Zanubrutinib

Based in NCCN clinical practice guidelines in oncology (NCCN Guidelines^®^) Antiemesis Version 1.2022

It is crucial to identify these factors when planning a cancer treatment strategy, given the high importance of avoiding CINV to achieve treatment goals and preserving patient’s quality of life. Various cancer scientific societies have developed and published guidelines on antiemetic therapy [5, 10–12] to provide tools to face the different risk scenarioswith the best prophylaxis of CINV.

Any cancer treatment should follow several principles for theprevention in CINV [13]:

- Prophylaxis is the primary goal of antiemetic therapy.
- Any patient or treatment with an emetic risk greater than 10% should receive adequate prophylaxis.
- Antiemetic therapy should cover the entire risk period.
- Oral or intravenous routes for antiemetic drugs offer the same efficacy.
- Selection of antiemetic therapy must be based on chemotherapy emetogenicity, plus patient’s risk factors.

## Guideline methods

The authors have reviewed the published clinical guidelines, as well as clinical trials from which the aspects referred to in these guidelines can be concluded.

Each author has been responsible for reviewing a part of the guideline that has been shared and discussed among all the authors to reach a consensus. Finally, the degrees of evidence and recommendation have been established based on the recommendations for the development of guidelines [14, 15].

## Types of chemotherapy-induced nausea and/or vomiting (CINV)

CINV is also known as emesis, although nausea and vomiting can occur independently due to their different pathophysiology [16, 17]. CINV is commonly classified into the following five types: acute, delayed, anticipatory, breakthrough, and refractory [18].

Acute CINV occurs in the first 24h after chemotherapy and its intensity peaks occur after 5–6h. In acute CINV, free radicals generated by chemotherapy stimulate cells in the gastrointestinal tract, which release serotonin (5-HT3). The activation of the 5-HT3 receptors triggers the vomiting reflex and chemoreceptor trigger zone in the central nervous system [19].

Delayed CINV occurs later than 24h after chemotherapy administration, typically between 48 and 72h and is mediated primarily by substance P; the action of substance P is mediated by NK1 receptor that affects sensory and nociceptive pathways and inflammation [5, 20]. It commonly occurs with administration of highly emetic chemotherapy; for cisplatin, delayed CINV can persist for 5–6days.

Anticipatory CINV occurs before receiving the next chemotherapy cycle. It is attributed to a previous adverse CINV experience, so it is considered a conditioned response. Incidence of anticipatory CINV ranges from 10 to 45%, and nausea is more common than vomiting. Significant predictive factors include younger age (< 50years), female gender and susceptibility to motion sickness [21, 22].

Breakthrough CINV occurs within 5days of the end of chemotherapy despite the use of adequate prophylactic antiemetic agents. This type of CINV usually requires rescue therapy with additional antiemetic treatment. Approximately 30–40% of patients receiving moderate or highly emetic chemotherapy can have breakthrough CINV and they should be considered for a higher level of prophylaxis during subsequent cycles of chemotherapy [22, 23].

Refractory CINV occurs in subsequent chemotherapy cycles despite the use of adequate antiemetic prophylaxis and rescue therapy.

## Overview and pharmacologic considerations

The pathophysiology of CINV involves the participation of various areas of the nervous system, as well as afferent and efferent pathways that will be responsible for emesis. Antiemetic drugs exert their action by acting on the receptors of the different neurotransmitters responsible for chemotherapy-induced emesis [24].

The dopamine D2 receptor antagonists (D2-RAs) include phenothiazine (proclorpromacine, perphenazine, and tietilperacilin), butyrophenones, (haloperidol and droperidol) and substituted benzamides (metoclopramide, domperidone, and alizapride). Currently, its use is relegated to refractory emesis or when modern agents or steroids are contraindicated (Level of Evidence V, Grade of Recommendation B).

The serotonin receptor antagonists (5-HT3-RAs) include first-generation agents—ondansetron, granisetron, dolasetron, tropisetron, and second-generation agents—palonosetron. Palonosetron has demonstrated greater efficacy than first-generation agents, as it produces a long-lasting serotonin receptor blockade and has synergistic activity with neurokinin inhibitors (Level of Evidence I, Grade of Recommendation A). Administration of these drugs days after chemotherapy is not recommended because it has not proven to be beneficial, and they have associated side effects.

The neurokinin-1 receptor antagonists (NK1-RAs) include aprepitant, fosaprepitant, and netupitant. Netupitant is a second-generation NK1-RA that targets the serotonin and substance P-mediated pathways involved predominantly in delayed emesis. Oral netupitant is combined with oral palonosetron (NEPA) in a single tablet [25, 26]. In combination with 5-HT3-RAs and steroids, NK1-RAs offer better control in acute and delayed emesis in highly emetic chemotherapy regimens (Level of Evidence I, Grade of Recommendation A).

Olanzapine is a second-generation antipsychotic agent that blocks serotonin 5-hydroxytryptamine (5-HT2) receptors and dopamine D2 receptors. A four-drug antiemetic regimen adding olanzapine is effective for preventing CINV in high emetic chemotherapy and moderate emetic chemotherapy schedules (Level of evidence I, Grade of Recommendation A) [27].

Current data indicate that dexamethasone doses may be individualized. High doses may be considered for non-NK1-RAs-containing regimens. Lower doses or shorter duration (“dexa sparing” schedules) can be used for non-cisplatin regimens based on patient characteristics (Level of Evidence I, Grade of Recommendation A) [28].

Other drugs such as benzodiazepines or cannabinoids have a different mechanism of action, and their use is controversial, generally relegated to delayed, anticipatory emesis or in the rescue treatment (Level of Evidence II, Grade of Recommendation C).

The combination of the different drugs, as stated in the guidelines, allows for antiemetic therapy to be adapted to each patient and clinical situation.

## Emesis prevention for high emetic risk IV anticancer drugs (Table 3)

Highly emetic chemotherapy (HEC) includes those agents or schedules that would cause emesis in more than 90% of the cases in the absence of antiemetic prophylaxis [24].

**Table 3:** Emesis prevention recommendations for high, moderate, low and minimal emetic risk IV anticancer drugs

	ASCO guidelines	NCCN guidelines	MASCC/ESMO guidelines
**Emesis prevention for high emetic risk IV anticancer drugs**			
ACUTE Day 1 (start before anticancer treatment)	*Single dose of NK1-RA (choose one):* Aprepitant 125 mg oral or 130 IV Fosaprepitant 150 mg IV Netupitant-palonosetron 300 mg netupitant/0.5 mg palonosetron oral in single capsule *Single dose of 5-HT3-RA (choose one):* Granisetron 2 mg PO / 1 mg IV once Ondansetron 8 mg PO or IV once Palonosetron 0.25 mg IV *Dexamethasone* 12 mg PO/IV *Olanzapine* 5-10 mg PO	OPTION A (PREFERRED) *Olanzapine* 5–10 mg PO once *Single dose of NK1-RA (choose one):* Aprepitant 125 mg oral Fosaprepitant 150 mg IV Netupitant-palonosetron 300 mg netupitant/0.5 mg palonosetron oral in single capsule *Single dose of 5-HT3-RA (choose one):* Granisetron 2 mg PO / 1 mg IV once Ondansetron 8–16 mg PO or 16–24 IV once Palonosetron 0.25 mg IV *Dexamethasone* 12 PO/IV OPTION B *Palonosetron* 0,25 mg IV once *Dexamethasone* 12 mg PO/IV *Olanzapine* 5-10 mg PO once OPTION C *Single dose of NK1-RA (choose one):* Aprepitant 125 mg oral Fosaprepitant 150 mg IV Netupitant-palonosetron 300 mg netupitant/0.5 mg palonosetron oral in single capsule *Single dose of 5-HT3-RA (choose one):* Granisetron 2 mg PO / 1 mg IV once Ondansetron 8–16 mg PO or 16–24 IV once Palonosetron 0.25 mg IV *Dexamethasone* 12 mg PO/IV once	*Single dose of NK1-RA (choose one) :* Aprepitant 125 mg oral Fosaprepitant 150 mg IV Netupitant-palonosetron 300 mg netupitant/0.5 mg palonosetron oral in single capsule *Single dose of 5-HT3-RA (choose one):* Granisetron 2 mg PO / 1 mg IV once Ondansetron 8 mg PO or IV once Palonosetron 0.25 mg iIV *Dexamethasone* 12 mg once *Olanzapine* 5–10 mg PO when nausea is an issue
DELAYED Days 2–4	*Aprepitant* 80 mg PO on days 2–3 (if aprepitant PO on day 1) *Dexamethasone* 8 mg oral or IV once daily on days 2-4 (*) *Olanzapine* 5–10 mg oral on days 2–4	OPTION A *Olanzapine* 5-10 mg PO daily on days 2-4 *Aprepitant* 80 mg PO daily on days 2,3 ( if aprepitant PO on day 1) *Dexamethasone* 8 mg PO/IV once daily OPTION B *Olanzapine* 5-10 mg PO daily on days 2-4 OPTION C *Aprepitant* 80 mg PO daily on days 2,3 (if aprepitant PO on day 1) *Dexamethasone* 8 mg PO/IV daily on days 2-4	*Aprepitant* 80 mg PO on days 2-3 (if aprepitant PO on day 1) *Dexamethasone* 8 mg once daily (*) *Olanzapine* 5–10 mg PO when nausea is an issue
**Emesis prevention for moderate emetic risk IV anticancer drugs**			
ACUTE Day 1 (start before anticancer treatment)	*Dexamethasone* 8 mg PO/IV once *Single dose of 5-HT3-RA (choose one):* Ondansetron 8 mg PO twice daily or 8 mg IV daily Palonosetron 0.25 mg iIV	*Dexamethasone* 12 PO/IV once *Single dose of 5-HT3-RA (choose one):* Granisetron 2 mg PO once or 1 mg IV Ondansetron 16-24 mg PO once or 8-16 IV once Palonosetron 0.25 mg IV once (preferred) +/− *Single dose of NK1-RA (choose one)* Aprepitant 125 mg PO once Fosaprepitant 150 mg IV once or Netupitant 300 mg/Palonsetron 0.5 mg PO once ALTERNATIVE TREATMENT *Olanzapine* 5–10 mg PO once *Palonosetron* 0.25 mg IV once *Dexametasone* 12 mg PO/IV once	*Dexamethasone 8 mg PO/IV once* *Single dose of 5-HT3-RA (choose one):* Granisetron 2 mg PO once or 1 mg IV Ondansetron 16–24 mg PO once or 8-16 IV once Palonosetron 0.25 mg IV once
DELAYED Days 2-3	No prophylaxis or Dexamethasone 8 mg PO or IV (**)	*Dexamethasone* 8 PO/IV once or *5-HT3-RA:* Granisetron 1–2 mg PO daily or 1 mg IV daily Ondansetron 8 mg PO twice daily or 16 mg PO daily or 8-16 mg IV daily OR Aprepitant 80 mg on days 2-3 (if oral aprepitant on day 1) PO +/- Dexamethasone 8 mg PO or IV daily on days 2 and 3 OR ALTERNATIVE TREATMENT *Olanzapine* 5-10 mg PO days 2-3	No prophylaxis or *Dexamethasone* 8 mg PO or IV daily (**)
**Emesis prevention for low emetic risk IV anticancer drugs**			
ACUTE Day 1 (start before anticancer treatment)	*Single dose of 5-HT3-RA (choose one):* Granisetron 2 mg PO or 1 IV once Granisetron 2 mg PO / 1 mg IV once Palonosetron 0.25 mg iIV OR *Dexamethasone* 8 mg PO/IV	*Dexamethasone* 8-12 PO/IV once OR *Metoclopramide* 10-20 mg PO/IV once OR *Prochlorperazine* 10 mg PO/IV once OR *5-HT3-RA (choose one):* Granisetron 1–2 mg (total dose) PO once Ondansetron 8–16 mg PO once	*Dexamethasone* 4–8 mg once OR *Single dose of 5-HT3-RA (choose one):* Ondansetron IV 8 mg or 8-16 mg PO Granisetron 1mg IV-PO or 2 mg PO Palonosetron 0.25 IV OR *Metoclopramide* 10–20 mg PO/IV once
DELAYED	No routine prophylaxis	No routine prophylaxis	No routine prophylaxis
**Emesis prevention for minimal emetic risk IV anticancer drugs**			
ACUTE Day 1	No routine prophylaxis	No routine prophylaxis	No routine prophylaxis
DELAYED	No routine prophylaxis	No routine prophylaxis	No routine prophylaxis

(*)Not recommended in Anthracycline combined with cyclophosphamide

(**)For agents known to cause delayed emesis (oxaliplatin, ciclofosfamide and doxorubicine)

HEC prophylaxis consists in administering a three-drug regimen including 5HT3-RAs, NK1-RAs and steroids (Level of Evidence I, Grade of Recommendation A) [2, 3]. Palonosetron is the preferred 5HT3-RA because of its superiority in controlling delayed emesis [29]. The addition of olanzapine to the triplet should be considered when the occurrence of nausea associated with HEC is an issue. (Level of Evidence I, Grade of Recommendation A). Data suggest that a 5mg dose of olanzapine is efficacious; this dose is recommended especially for elderly or oversedated patients [27, 30].

Efficacy of the three-drug antiemetic regimen olanzapine plus palonosetron plus dexamethasone did not differ significantly in terms of complete response rates to aprepitant plus palonosetron plus dexamethasone. (Level of Evidence IB, Grade of Recommendation A) [4].

Current data indicate that the dexamethasone doses may be individualized. High doses may be considered for no-NK1 regimens. Low doses or with shorter duration can be planned for non-cisplatin regimens based on patient characteristics. (Level of evidence 2, Grade of Recommendation A) [11, 12].

## Emesis prevention for moderate emetic risk IV anticancer drugs (Table 3)

Moderately emetic chemotherapy (MEC) includes those with an associated risk of CINV between 30 and 90%. The combination of a 5-HT3-RA and dexamethasone is the preferred option for the prevention of acute emesis [5, 11, 18]. The 5-HT3-RA of choice is palonosetron [31]. (Level of Evidence II, Grade of Recommendation B). There are other alternatives to prevent acute emesis in MEC, such as the addition of a NK1-RA to 5-HT3-RA and dexamethasone or the combination of olanzapine, palonosetron and dexamethasone. (Level of Evidence II, Grade of Recommendation B).

To prevent carboplatin-induced acute emesis, a combination of an NK1-RA, 5-HT3-RA and dexamethasone is recommended (Level of Evidence II, Grade of Recommendation B) [32].

Prophylactic treatment against delayed CINV is not routinely recommended in patients receiving MEC except with those therapies that are more frequently associated with the onset of delayed CINV (for example, oxaliplatin, anthracyclines or cyclophosphamide). In these cases, it is recommended to add dexamethasone on days 2 and 3. Other alternatives are the use of olanzapine or 5-HT3-RA on days 2 and 3 (Level of evidence II; Grade of Recommendation B).

## Emesis prevention for low and minimal emetic risk IV anticancer drugs (Table 3)

Drugs with low emetic potential are those for which the risk of CINV lies between 10 and 30%. For drugs having a minimal emetogenic potential, the risk is < 10%. Most new targeted agents and immune-checkpoint inhibitors are included in this category. The optimal treatment to prevent acute CINV in patients receiving low emetic risk anticancer drugs (CT) includes a single antiemetic agent administered before treatment such as 5-HT3-RA, dexamethasone or D2-RAs, (i.e., metoclopramide) (Level of Evidence II, Grade of Recommendation B) [5]. The use of antiemetic prophylaxis against delayed CINV for low emetogenic CT is not recommended (Level of Evidence II, Grade of Recommendation B) [11].

No antiemetic treatment should be routinely administered before or after minimally emetogenic antineoplastic agents in patients without a history of nausea and vomiting. (Level of Evidence IV, Grade of Recommendation D). If a patient experiences acute or delayed nausea or vomiting after low or minimally emetogenic drug, prophylactic antiemetic treatment might be considered for subsequent chemotherapy administrations using the regimen for the next higher emetic level (Level of Evidence II, Grade of Recommendation B) [9, 18].

## Emesis prevention for multiday IV chemotherapy

Prophylaxis of CINV in patients receiving moderately or highly emetic multiday chemotherapy is more difficult, due to a mixture of acute and delayed effects, as well as anticipatory emesis [18]. Practical issues should be considered (i.e., route of administration, administration setting or duration of action of 5-HT3-RA, dosing intervals, compliance issues or individual risk factors). Moreover, there are few clinical studies that look at this situation [11].

Patients receiving moderately or highly emetic multiday chemotherapy should receive a 5-HT3-RA plus dexamethasone for acute emesis and dexamethasone for delayed emesis (Level of Evidence II, Grade of Recommendation A). Dexamethasone should be administered once daily in the morning and maintained 2–3days after chemotherapy for regimens likely to cause significant delayed emesis [11]. NK1-RA may also be used in those regimens associated with significant risk of delayed emesis. If the regimen does not contain an NK1-RA, palonosetron is the preferred 5-HT3-RA [33]. For patients with moderately emetic multiday chemotherapy, limiting the administration of dexamethasone to day 1 is an option (especially in intolerant to corticosteroids patients) that may not be associated with a significant efficacy reduction [11]. If patients cannot tolerate dexamethasone, consider replacing with olanzapine.

## Emesis prevention for oral anticancer drugs

Separate classifications have been established for intravenous and oral antineoplastic agents (oral agents are usually given daily and over longer periods). This classification has some limitations: categorical data on the intrinsic emetic risk are available for only few agents, the classification underestimates the risk of delayed emesis and of acute and delayed nausea, and the classification does not address the emetogenic potential of combination regimens, which is usually determined by the most emetic agent of the combination [24]. Numerous new oral antineoplastic agents have been introduced in NCCN and MASCC/ESMO antiemetic guideline update that must be incorporated into the emetogenic classification [11, 18].

Oral antiemetic prophylaxis is recommended for highly or moderately emetogenic oral agents. Single-agent antiemetic therapy with 5HT3-RA should be started before anticancer therapy and continue daily for the duration of the treatment (Level of Evidence II, Grade of Recommendation B) [18, 34].

Recommended prophylaxis in patients receiving low- or minimal-emetogenic oral agents with a single antiemetic oral agent like a D2-RA (metoclopramide, prochlorperazine) or a 5HT3-RA is recommended in case of appearance of CINV. Level of Evidence II, Grade of Recommendation B). If multiple oral agents are combined, emetic risk may increase and require adequate prophylaxis.

## Breakthrough emesis and rescue antiemetic therapy

Breakthrough nausea and vomiting and rescue antiemetic therapy is a challenging situation. Other causes for emesis (i.e., use of opioid medication, central nervous system metastases, hypercalcemia, or gastrointestinal obstruction) must be ruled out. There are no data from specifically designed clinical trials in this area (Level of Evidence V, Grade of Recommendation C), but the following recommendations can be followed [35, 36]:

- Patients must have received appropriate antiemetic treatment (Level of Evidence I, Grade of Recommendation A).
- There is evidence to suggest that refractory emesis may respond to a switch from one 5-HT3-RA to another (Level of Evidence II, Grade of Recommendation C).
- After a course refractory to antiemetic treatment, an attempt can be made to adjust the scheme for the next cycle to a higher risk group (Level of Evidence V, Grade of Recommendation C).
- If the patient received an oral regimen, the physician could consider giving agents intravenously, although there is no evidence that this will improve efficacy (Level of Evidence V, Grade of Recommendation C).
- As rescue therapy, a drug with a different mechanism of action can be used (i.e. lorazepam, alprazolam, olanzapine, prochlorperazine, or haloperidol) (Level of Evidence V, Grade of Recommendation C). Olanzapine has shown superiority over metroclopramide in a recent randomized trial (Level of Evidence II, Grade of Recommendation B).

## Anticipatory emesis prevention and treatment

The best way to prevent anticipatory nausea and vomiting is to achieve good control of acute and delayed emesis (Level of evidence III; Recommendation grade B) [37, 38]. Benzodiazepines may help in the treatment of anticipatory nausea, as they help reduce the anxiety associated with chemotherapy administration and the most studied therapy is lorazepam [39, 40]. The recommended regimen of lorazepam is to start at doses of 0.5–2mg the night before antitumor treatment and repeating the dose 1 or 2h before administration (Level of evidence II; Grade of Recommendation A). Other therapies such as acupuncture [41, 42] have been shown to be effective in controlling anticipatory emesis (Evidence level II; Recommendation grade B). Behavioral interventions, such as progressive muscle relaxation and systematic desensitization training, should be considered effective methods for the prevention and treatment of this type of emesis (Evidence level II, Recommendation grade B) [43, 44].

## Radiation induced emesis prevention

Radiation-induced emesis (RINV) is divided into four risk levels: high, moderate, low, and minimal. These levels depend on the site of radiation and do not consider radiation dose, fractionation, technique or other proposed risk factors [5].

Patients on highly emetogenic radiotherapy (total body irradiation) should receive an oral 5-TH3-RA ± dexamethasone each day of radiotherapy treatment. (Level of Evidence II, Grade of Recommendation B).

Subjects receiving moderately emetic radiotherapy (upper abdomen, craniospinal locations) should receive an oral 5-HT3-RA each day of treatment and optional short-course of oral dexamethasone (Level of Evidence II, Grade of recommendation A) [11].

Those who are receiving low emetic radiotherapy (thorax, cranium, head and neck, and pelvis) should receive prophylaxis or rescue with one of the following drugs: an oral 5-HT3-RA, dexamethasone (preferred in SNC RT), or a D2-RA (Level of Evidence IV, Grade of Recommendation D) [9].

Patients receiving minimally emetic radiotherapy (extremities, breast) should not receive antiemetics routinely, but breakthrough treatment with a D2-RA or a 5-HT3-RA may be prescribed if patient presents radiation-induced emesis (Level of Evidence IV, Grade of Recommendation D) [18].

## Concurrent chemoradiotherapy-induced emesis prevention

In patients receiving concurrent chemoradiotherapy, it is advised to prescribe antiemetics according to the emetogenic potential of the chemotherapy unless it is considered that the risk of nausea and vomiting induced by the radiotherapy is higher.

During periods when prophylactic antiemetic therapy for chemotherapy has ended and ongoing radiotherapy continues, patients should receive prophylactic therapy appropriate for the emetogenic risk of the radiotherapy until the next course of chemotherapy, rather than receiving breakthrough therapy (Level of Evidence II, Grade of recommendation A) [45, 46].

## Summary of recommendations

**Table:** 

Principles of prevention in CINV	Prophylaxis is the primary goal of antiemetic therapy Any patient receiving treatment with an emetic risk greater than 10% should receive adequate prophylaxis Antiemetic therapy should cover the entire risk period Oral or intravenous routes for antiemetic drugs offer the same efficacy Selection of antiemetic therapy must be based on chemotherapy emetogenicity and patient’s risk factors
Emesis prevention in high emetic risk IV anticancer drugs	HEC prophylaxis consists in a three-drug regimen including 5-HT3-RAs, NK1-RA and steroids Palonosetron is the preferred 5HT3-RA The addition of olanzapine should be considered when the occurrence of nausea is an issue A three-drug antiemetic regimen with olanzapine, palonosetron and dexamethasone is an alternative
Emesis prevention in moderate emetic risk IV anticancer drugs	A combination of 5-HT3-RA and dexamethasone is the preferred option to prevent acute emesis Palonosetron is the preferred 5HT3-RA The addition of NK1-RA or olanzapine can be considered A combination of a NK1-RA, 5-HT3-RA and dexamethasone is recommended to prevent carboplatin induced acute emesis Prophylaxis against delayed emesis is not routinely recommended MEC except with those therapies frequently associated with delayed CINV
Emesis prevention in low and minimal emetogenic risk IV anticancer drugs	A single antiemetic agent such as 5-HT3-RA, dexamethasone or a D2-RA is recommended to prevent acute emesis with low emetogenic agents Prophylaxis against delayed emesis is not routinely recommended with low and minimal emetogenic agents If patients experience CINV after low or minimally emetogenic drug, prophylactic antiemetic treatment might be considered for subsequent cycles using the regimen for the next higher emetic level
Emesis prevention in multiday chemotherapy	Patients receiving HEC or MEC multiday chemotherapy should receive a 5-HT3-RA plus dexamethasone for acute emesis and dexamethasone for delayed emesis NK1-RA may also be used regimens with significant risk of delayed emesis If NK1-RA is not included, palonosetron is the preferred serotonin antagonist is 5HT3-RA
Emesis prevention in oral anticancer drugs	Prophylaxis with daily treatment with oral 5HT3-RA is recommended in patients receiving oral HEC or MEC Prophylaxis with daily treatment with oral D2-RA is recommended in patients receiving oral low- or minimal-emetogenic oral
Breakthrough emesis and rescue antiemetic therapy	After a course refractory to antiemetic treatment, adjust the scheme for the next cycle to a higher risk group As rescue therapy, a drug with a different mechanism of action can be used such as Olanzapine or benzodiazepines
Anticipatory emesis prevention and treatment	The best way to prevent anticipatory emesis is to achieve good control of acute and delayed emesis Benzodiazepines may be helpful, as they help reduce the associated anxiety
Radiation-induced emesis prevention	Prophylaxis with oral 5-TH3-RA ± dexamethasone daily is recommended in patients on HEC Prophylaxis with oral 5-TH3-RA ± short course of dexamethasone daily is recommended in patients on moderately emetogenic radiotherapy Patients receiving low emetic radiotherapy should receive prophylaxis or rescue with oral 5-HT3-RA, dexamethasone (preferred in SNC RT), or a D2-RA Prophylaxis in patients receiving minimally emetic radiotherapy is not routinely recommended
Concurrent chemoradiotherapy	Patients should receive antiemetic treatment according to the emetogenic potential of the chemotherapy unless the risk of emesis induced by the radiotherapy is higher
