# Correction to: SEOM Clinical Guideline update for the prevention of chemotherapy-induced nausea and vomiting (2021)

**DOI:** 10.1007/s12094-022-02846-3

**Published:** 2022-05-14

**Authors:** Margarita Majem, Ramon de las Peñas, Juan Antonio Virizuela, Luís Cabezón-Gutiérrez, Patricia Cruz, Rafael Lopez-Castro, Miriam Méndez, Rebeca Mondéjar, María del Mar Muñoz, Yolanda Escobar

**Affiliations:** 1 Department of Medical Oncology, Hospital de la Santa Creu i Sant Pau, c/Sant Antoni Maria Claret 167, 08025 Barcelona, Spain; 2 Department of Medical Oncology, Hospital Provincial Castelló, Valencia, Spain; 3 Department of Medical Oncology, Hospital Universitario Virgen Macarena, Seville, Spain; 4 Department of Medical Oncology, Hospital Universitario de Torrejón, Madrid, Spain; 5 Department of Medical Oncology, Hospital Universitario La Paz, Madrid, Spain; 6 Department of Medical Oncology, Hospital Clínico Universitario de Valladolid, Valladolid, Spain; 7 Department of Medical Oncology, Hospital Universitario Puerta de Hierro-Majadahonda, Madrid, Spain; 8 Department of Medical Oncology, Hospital Universitario de La Princesa, Madrid, Spain; 9 Department of Medical Oncology, Hospital Virgen de la Luz de Cuenca, Cuenca, Spain; 10 Department of Medical Oncology, Hospital General Universitario Gregorio Marañón, Madrid, Spain

## Correction to: Clinical and Translational Oncology (2022) 24:712–723 10.1007/s12094-022-02802-1

In this article the title was incorrectly given as “SEOM clinical guideline emesis (2021)” but should have been “SEOM Clinical Guideline update for the prevention of chemotherapy-induced nausea and vomiting (2021).”

The original article has been corrected.

